# SIV reservoir depletion and clearance from neonatal macaques following transient CCR5 depletion

**DOI:** 10.1038/s41564-026-02511-3

**Published:** 2026-10-19

**Authors:** Jesse D. Deere, David Merriam, Kawthar Machmach, W. L. William Chang, Michael Nekorchuk, Gema Méndez-Lagares, Hung Kieu, Joseph Dutra, Justin Fontaine, Denise R. Andal, Kateland Sipe, Elizabeth Heninger, Margaret Martin, Wenze Lu, Ning Chin, Connie Chen, Bryant Chi-Thien Tran, Jessica Salinas, Corey N. Miller, Steven G. Deeks, Jeffrey D. Lifson, Kathleen Engelman, Diogo Magnani, Keith Reimann, Mario Stevenson, Jacob D. Estes, Dennis J. Hartigan-O’Connor

**Affiliations:** 1 Department of Medical Microbiology and Immunology, School of Medicine, University of California Davis, Davis, CA USA; 2 National Biomedical Research Institute, University of California Davis, Davis, CA USA; 3 The US Military HIV Research Program, Walter Reed Army Institute of Research, Silver Spring, MD USA; 4 Henry M. Jackson Foundation for the Advancement of Military Medicine, Bethesda, MD USA; 5 Vaccine and Gene Therapy Institute, Oregon Health and Science University, Beaverton, OR USA; 6 Department of Medicine, University of Miami Miller School of Medicine, Miami, FL USA; 7 Division of Infectious Diseases, University of Miami Miller School of Medicine, Miami, FL USA; 8 Diabetes Center, University of California San Francisco, San Francisco, CA USA; 9 Department of Medicine, University of California San Francisco, San Francisco, CA USA; 10 AIDS and Cancer Virus Program, Frederick National Laboratory for Cancer Research, Frederick, MD USA; 11 Nonhuman Primate Reagent Resource, UMass Chan Medical School, Worcester, MA USA; 12 Oregon National Primate Research Center, Oregon Health and Science University, Beaverton, OR USA; 13 Division of Experimental Medicine, Department of Medicine, University of California San Francisco, San Francisco, CA USA

**Keywords:** HIV infections, HIV infections

## Abstract

Antiretroviral therapy (ART) suppresses viraemia in people with human immunodeficiency virus (HIV), but HIV provirus persists indefinitely in CD4-expressing cells. Cells containing active provirus express viral proteins and have been successfully targeted using broadly neutralizing antibodies (bnAbs). In contrast, cells containing latent provirus cannot be specifically targeted without latency reactivation. Meaningful depletion of the latent HIV or simian immunodeficiency virus (SIV) reservoirs has not been demonstrated except with cytotoxic conditioning for bone-marrow transplantation. Here we show that both the active and latent SIV reservoirs can be eliminated from infant macaques via profound but transient depletion of cells that express CCR5 using a single shot of CCR5/CD3 bispecific antibody. Neonatal rhesus macaques were infected with SIVmac251, then treated with ART beginning at 1 week after infection, followed by treatment with either a CCR5/CD3-bispecific or a CD4-specific antibody, both of which depleted target cells and accelerated the decline in plasma viraemia. Upon ART cessation, while rebound occurred quickly in all animals treated with ART alone, most animals treated with CCR5/CD3 bispecific antibody remained aviraemic for >3 months. Several remained aviraemic to the end of follow-up and efforts to detect replication-competent virus were unsuccessful. Our results show that bispecific antibody treatment achieves meaningful depletion of the replication-competent SIV reservoir and supports the possibility that clinically meaningful depletion of the HIV reservoir might be achievable for recently infected individuals.

## Abstract (web summary)

Transiently depleting cells that express the HIV co-receptor, CCR5, using a CCR5/CD3 bispecific antibody after stopping antiretroviral therapy in monkeys maintains them virus-free for at least several months.

## Main

Instances of complete human immunodeficiency virus (HIV) clearance or remission have been rare. Notable cases of apparent HIV cure have involved people with HIV (PWH) who were treated aggressively for underlying malignancies with regimens that included radiation and/or cytotoxic chemotherapy or immunotherapy and bone marrow transplantation from donors with a mutation in chemokine receptor 5 (CCR5Δ32/Δ32), known to provide resistance to HIV replication^1–7^. The results suggest that elimination of cells bearing this chemokine receptor can make an important contribution to eradication of the virus.

Antiretroviral therapy (ART) does not cure HIV or simian immunodeficiency virus (SIV) infections once a reservoir has been fully established, within ~3 days after infection^8–14^. No instance of apparent cure was seen when ART was started at 3 or 7 days after SIV or SHIV-SF162P3 infection (0/8 and 0/11 in refs. ^15^ and ^16^, respectively). In another example, 39 animals were infected intravenously with two focus-forming units of SIVmac239X^17^, an inoculum that models HIV transmission to humans and was predicted to result in slow spread of the virus, and were then treated with ART beginning at 4–12 days after infection. In this study, as well, no cure was demonstrated when ART was started at 7 days after infection (0/10) and only 1 of 13 animals treated at 6 days after infection failed to rebound. In an early-treatment model employing the low-fitness transmitted founder-based SHIV, SHIV.C.CH505 (refs. ^18–20^), it was shown that peak plasma viral load before ART was the most important correlate of time to rebound^21^. SIV infection of Mauritian cynomolgus macaques can be cured by allogeneic stem-cell transplantation in some cases, but clearance is sometimes prevented by viral spread to engrafting CCR5^+^ donor cells despite ongoing suppressive ART^22^.

Proposed approaches to latent reservoir reduction include gene editing, ‘block-and-lock’ or latency reversal combined with immunologic targeting of reactivated cells^23^. One approach is to combine latency reactivation with broadly neutralizing antibodies^16,24–26^, which have recently proven dramatically effective in suppressing the active SHIV reservoir and providing functional cure of infant macaques^27^. Direct depletion of the latent and active reservoirs (together, the replication-competent viral reservoir or RCVR) has received comparatively little attention. As there is no specific marker for latently infected cells, selective depletion of those cells is not possible. However, elimination of host immune cells that encompass the RCVR could prove effective and safe. Two previous studies showed that depletion of CD4^+^ cells relatively late in infection (7–18 months) produced a significant reduction in peripheral blood mononuclear cell (PBMC)-associated SIV genomes^28^ as well as trends to a reduction in SIV outgrowth^28^ and to extended rebound times^29^. Another demonstrated that elimination of more than ~55% of CCR5-expressing cells was sufficient for benefit as measured by resistance to challenge and post-infection viral load^30^.

In this study, we tested the hypothesis that early depletion of most infected cells and those which might be susceptible to new infection events could prove curative. We chose to test this hypothesis soon after infection when the RCVR remains limited. We also chose to initially treat infants because they have the capacity for robust, long-term T cell reconstitution due to active thymic output.

## SIV target-cell depletion after perinatal infection

**Fig. 1:**
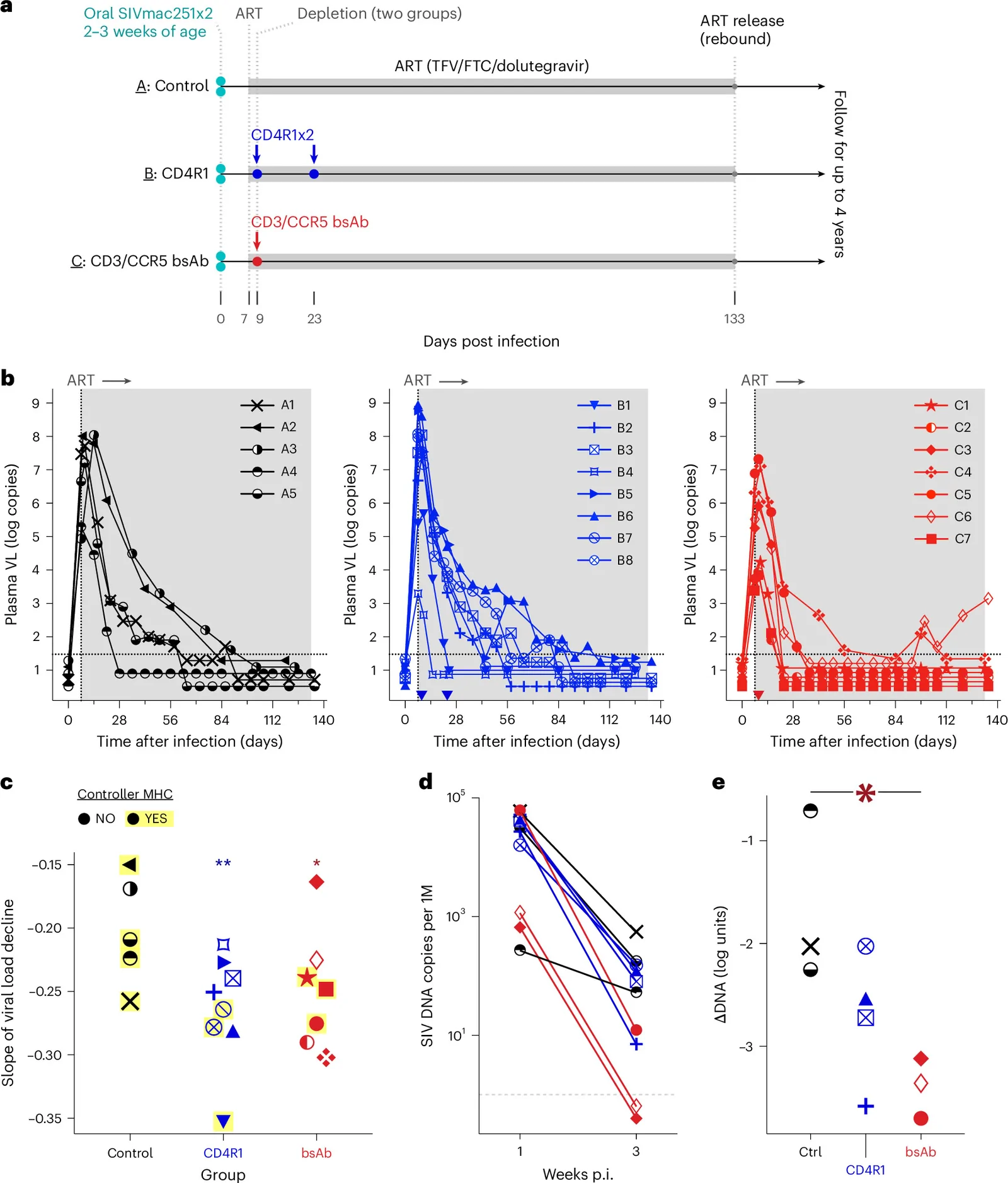
SIV infection of newborn macaques, early treatment and depletion of potential reservoir cells. **a**, Experimental schema. Macaques were randomized to experimental treatment groups at birth. To model perinatal infection, they were then infected at 2 weeks of age by oral inoculation twice on the same day. Antiretroviral therapy with tenofovir (TFV), emtricitabine (FTC) and dolutegravir was started at 7 days after infection. Depleting antibodies were given at 2 and 16 days (group B; CD4R1) or only at 2 days (group C, anti-CD3/CCR5 bsAb) after ART initiation. Therapy was withdrawn at 19 weeks (133 days) after infection. **b**, Plasma SIV RNA viral loads (VL) in the infected macaques of groups A, B and C, respectively, before treatment interruption. **c**, The slope of viral-load decline in each animal was calculated by linear regression using the peak value and those from the subsequent 3 weeks (asterisks indicate *P* < 0.05 for contrast of this group with the control group; **P = 0.0089, *P = 0.0338). **d**, Cell-associated SIV DNA copies assessed before and after depletion. **e**, The log fold-change in SIV DNA copies in each macaque between 1 and 3 weeks post infection, calculated using the values in **d**. Linear regression of the log-fold changes vs group assignment showed greater SIV DNA depletion in the bsAb recipients of group C than in controls (*P* = 0.01). Source data

**Extended Data Fig. 1:**
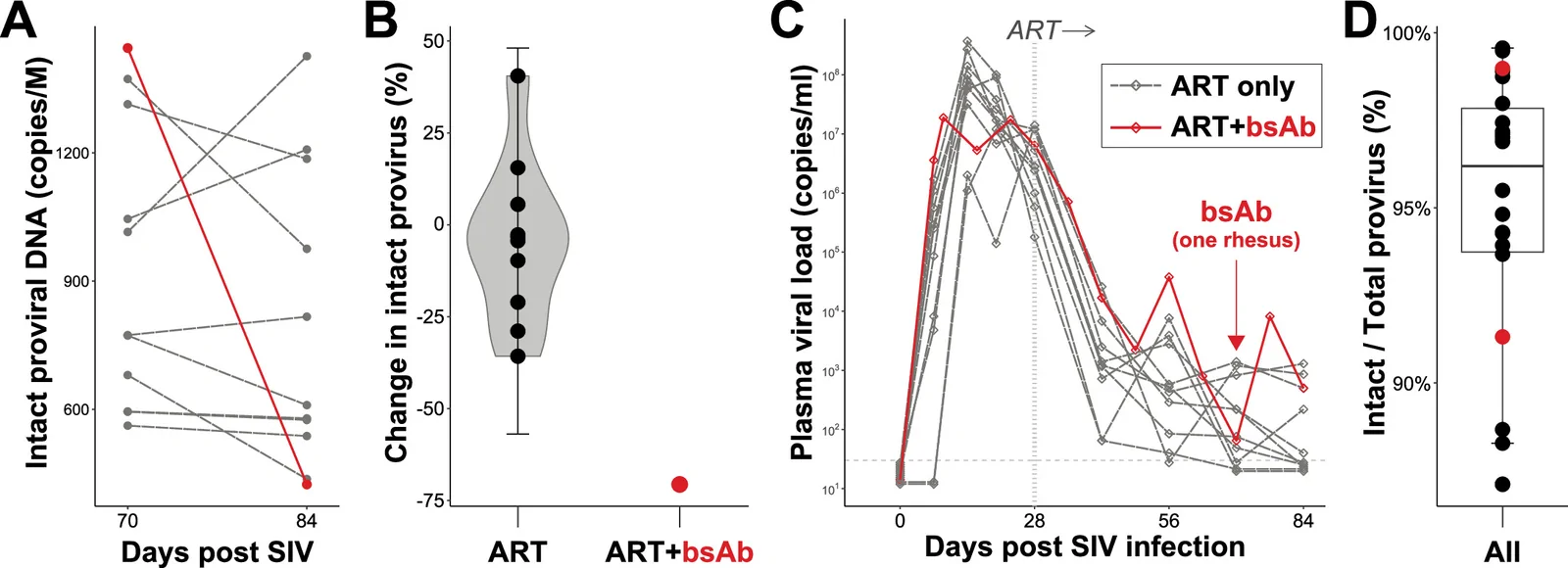
CD3/CCR5 bispecific antibody (bsAb) can deplete reservoir cells when administered 70 days post infection. **(A)** 11 macaques were infected with SIVmac251 and ART treatment was initiated 28 days post infection. CD3xCCR5 bispecific antibody (bsAb) was administered to one animal 70 days after infection. Intact proviruses were assessed using the IPDA assay (Accelevir) at 70 and 84 days, which were time points before and after bsAb treatment. Only the bsAb-treated macaque demonstrated significant reservoir depletion. 3.8-9.6 × 10^5^ cells were used for each assay. **(B)** Change in frequency of intact proviruses. bsAb treatment was associated with the greatest loss of proviral DNA, which exceeded both the 95% prediction interval (plotted) and 98% prediction interval (-69.9% to +61.1%) established by the 10 untreated animals. **(C)** Viral-load kinetics in the treated animal and controls. **(D)** A mean of 95% of total proviruses detected were found to be intact (range in 22 samples, 87.1 to 99.6%). Boxplots display the median value, lower hinge at the first quartile, upper hinge at the third quartile, lower whisker at the smallest value no further than 1.5 times the inter-quartile range (IQR) from the lower hinge, and upper whisker at the largest value no further than 1.5 times the IQR from the upper hinge. Source data

We previously reported development of an anti-CCR5/CD3 bispecific antibody (bsAb) able to deplete CCR5-expressing cells from blood and gut tissue of SIV-naïve macaques^31^. To test the concept that depleted CCR5^+^ cells might include established SIV reservoir cells, we followed intact proviruses in the blood cells of one SIV+ macaque receiving bsAb at 10 weeks after infection (after 6 weeks of ART). We observed 71% reservoir depletion 2 weeks after bsAb administration, which exceeded the change seen in any of 10 controls (Extended Data Fig. 1) and exceeded the lower threshold in ref. ^30^, but also suggested that earlier treatment of smaller reservoirs would more likely be clinically beneficial. We therefore pursued reservoir depletion in the context of an early-treatment protocol modelling perinatal infection followed by early ART, with attempted reservoir depletion soon thereafter (Fig. 1a). The two depleting agents tested were CCR5/CD3 bsAb and CD4R1 antibody, which targeted either CCR5- or CD4-expressing cells, respectively. To maximize signal for effects that might prove clinically meaningful, we chose high-dose oral infection with SIVmac251, a highly pathogenic heterogeneous swarm^32,33^. Neonatal macaques that had been randomized to experimental groups on the day of collection were inoculated at ~2–3 weeks of age with 50,000 TCID_50_ (50% tissue culture infectious dose) of SIVmac251, given orally twice on the same day. The inoculated animals were maintained untreated for 7 days to permit establishment of a permanent and systemic infection before the initiation of ART (tenofovir, emtricitabine and dolutegravir^34^). Two days after ART initiation, group A received only continued ART; group B received CD4R1; and group C received a single shot of anti-CD3/CCR5 bsAb. ART was withdrawn after 18 weeks and viral rebound monitored.

**Fig. 2:**
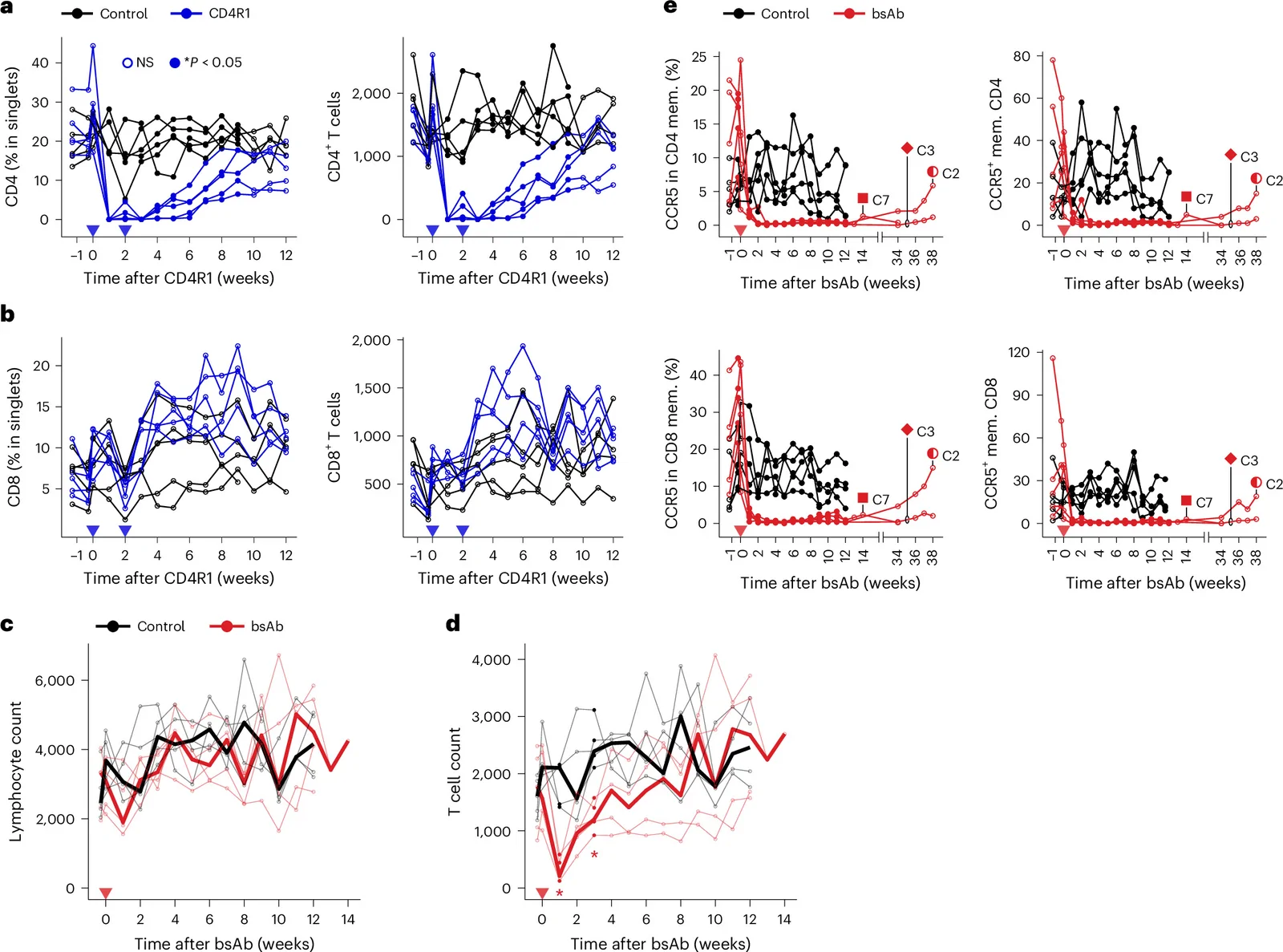
Effects of CD4R1 or CCR5/CD3 bsAb on lymphocyte subsets. **a**, In control animals and CD4R1 recipients, CD3^+^CD4^+^ T cells detected by flow cytometry, as a percentage of singlet lymphocytes (left) or absolute counts per ml of blood (right). Linear regression was performed at each time point using the percentage values as the dependent variable and group membership as the independent variable; plotted markers are filled when the adjusted *P* value for that time point was <0.05. The same procedure was used for all graphs in **a**–**e**. Data for 15 animals are shown in this figure and the remaining data relevant to depletion, collected on a different schedule, in Extended Data Fig. 5. **b**, CD3^+^CD8^+^ T cells detected by flow cytometry, as a percentage of singlet lymphocytes or absolute counts. **c**, In CCR5/CD3 bsAb recipients, lymphocyte counts are not changed by bsAb treatment. **d**, CD3^+^ T cell counts, demonstrating a temporary reduction in the weeks after bsAb administration. Statistical comparisons were performed at each time point using two-sided *t*-test (via ordinary least squares regression). To account for multiple comparisons across the sequence of time points, *P* values were adjusted according to Benjamini and Hochberg (*P = 0.0005 at 1 week and 0.0020 at 3 weeks)^84^. **e**, CCR5 expression as a percentage of CD3^+^CD4^+^CD95^+^ memory T cells (top left; evaluated using a non-cross blocking mAb to CCR5); absolute counts of CCR5^+^ memory CD4^+^ T cells (top right). CCR5 expression as a percentage of CD3^+^CD8^+^CD95^+^ memory T cells (bottom left); absolute counts of CCR5^+^ memory CD8^+^ T cells (bottom right). Source data

Twenty of 25 challenged infants were infected by the oral inocula (Fig. 1b and Extended Data Table 1), with 3/5 uninfected infants having inherited a Mamu allele associated with post-acquisition control of viral replication. All the infected animals developed unambiguous systemic SIV infection, characterized by both (1) ≥2 consecutive viral loads over the limit of detection of our assay (Fig. 1b) and (2) detection of cell-associated SIV RNA (caRNA) at least twice, ≥8 weeks post infection, in every macaque tested for caRNA including 3/3 late-rebounding recipients of CD3/CCR5 bsAb.

Compared with those treated with ART alone, both groups of antibody-treated macaques experienced more rapid viral-load decay after treatment (Fig. 1c; *P* = 0.009 and 0.03 for slope of decay in groups B and C), suggesting either a rapid effect of depletion or a secondary effect on SIV spread, such as blockade of viral entry^35^. Mamu allele inheritance did not predict viral-load decay or time to aviraemia.

We tracked the amount of cell-associated SIV DNA before and after antibody treatment in newborns from which sufficient cells were obtained (Fig. 1d,e). We observed an average of 1.7 log units of depletion in cell-associated DNA in ART-only control macaques over this time, 2.7 units in CD4R1 recipients and 3.4 log units of depletion in bsAb recipients (*P* = 0.01 for better depletion in group C). Of the total proviruses, 95% are intact during this early phase of SIV infection (Extended Data Fig. 1d).

## Long-lasting effect of bispecific antibody on blood and tissue immunophenotypes

**Extended Data Fig. 2:**
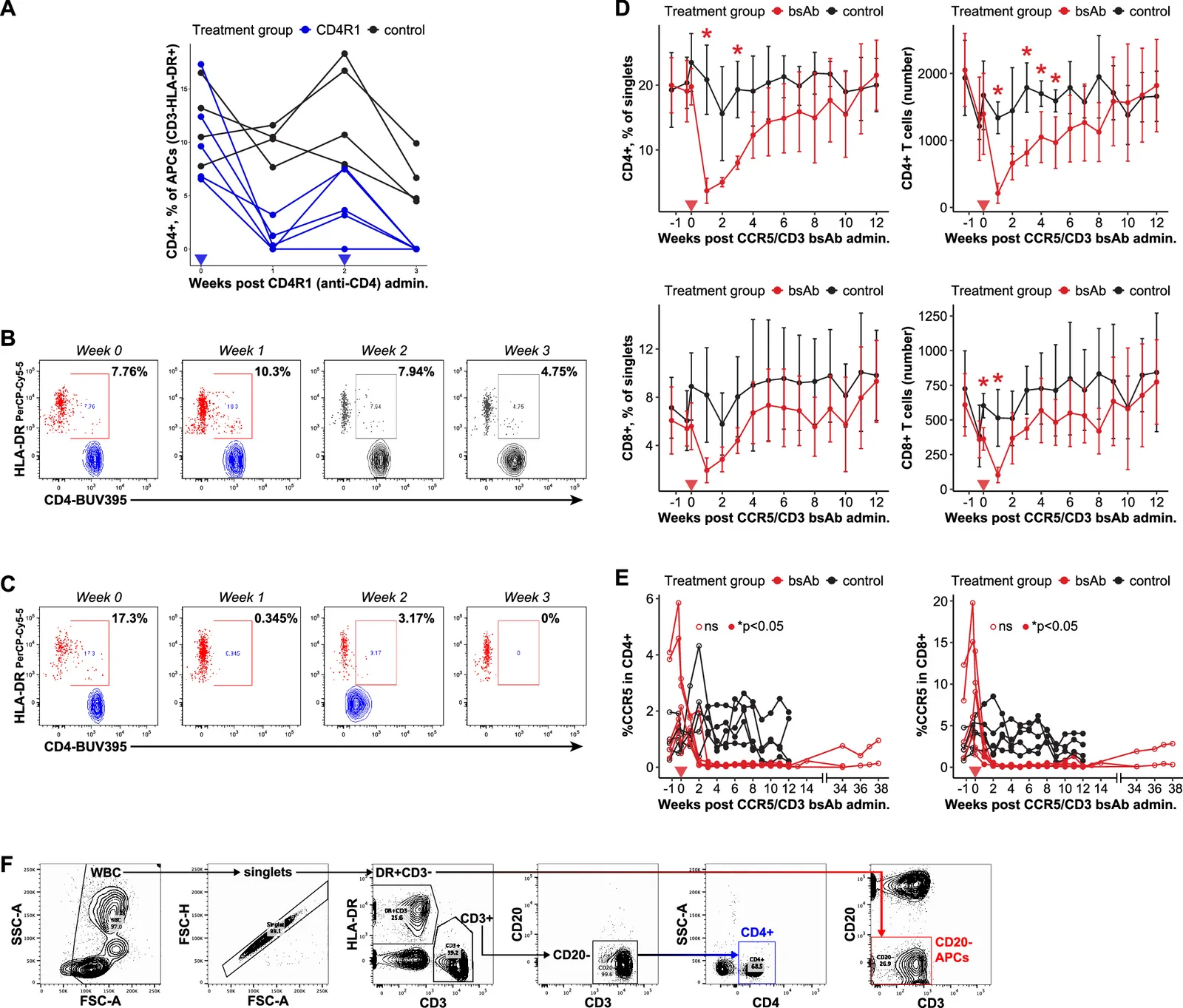
Dynamics of CD4^+^ APCs in Group B and CD4^+^/CD8^+^ T cells in Group C. **(A)** After a first administration of depleting antibody (week 0), representation of CD4-expressing cells among APCs declines precipitously. After a second administration (week 2), depletion of CD4^+^ APCs is complete. **(B)** Maintenance of CD4 expression by the APCs of a control (undepleted) animal. **(C)** Complete depletion of CD4-expressing APCs by the fourth week after anti-CD4 antibody administration. **(D)** Treatment with CD3/CCR5 bsAb in Group C was associated with only transient CD4/8 deficiency during the period of T-cell lymphopenia (Fig. 2D). Data are presented as mean values +/- SD (n = 5 per group). Statistical comparisons were performed at each time point using two-sided t-tests (via ordinary least squares regression). To account for multiple comparisons across the sequence of time points, p-values were adjusted according to Benjamini and Hochberg (*p = 0.0022 and 0.0035 at 1 and 3 weeks at upper left; 0.0003, 0.0059, 0.0388, and 0.0388 at weeks 1 and 3-5, respectively, at upper right; and 0.0280 at weeks 0 and 1 at lower right). **(E)** CCR5 expression as a percentage of total CD3^+^CD4^+^ T cells (left); CCR5 expression as a percentage of total CD3^+^CD8^+^ T cells (right). Differences between treatment groups at each time point using two-sided Wilcoxon rank-sum tests, unadjusted for multiple comparisons. **(F)** Gating strategy for the flow cytometry. Source data

Following antibody treatment, animals in group B (CD4R1) exhibited a profound depletion of CD4^+^ cells from blood, including ~100% of CD4^+^ monocytes (Extended Data Fig. 2a–c) and ~100% of CD4^+^ T cells^28,29,36,37^ (Fig. 2a). This depletion was durable, with a substantial deficiency of such cells persisting in 2/5 animals until 12 weeks after the first antibody administration (Fig. 2a). CD8^+^ T cells were not depleted (Fig. 2b). Animals in group C (CCR5/CD3 bsAb) first exhibited temporary CD3^+^ lymphopenia (Fig. 2c,d) and then specific, durable loss of CCR5^+^ cells in agreement with the pattern we previously described^31^ (Fig. 2e). Loss of circulating CCR5^+^ cells from blood was essentially complete, with ≤3 CCR5^+^ CD4^+^ memory and ≤5 CCR5^+^ CD8^+^ memory T cells detected in all treated animals for at least 14 weeks post bsAb administration (Fig. 2e, right panels showing absolute counts per ml of blood). Using the same bsAb, we previously demonstrated depletion of CCR5^+^ cells from lymph node (LN) and colon^31^, so the depletion we observed in blood was not caused by trafficking of CCR5^+^ cells to those tissues. The fraction and numbers of circulating CD4^+^ and CD8^+^ T cells remained normal in group C, except during the period of CD3^+^ lymphopenia (Extended Data Fig. 2d).

**Extended Data Fig. 3:**
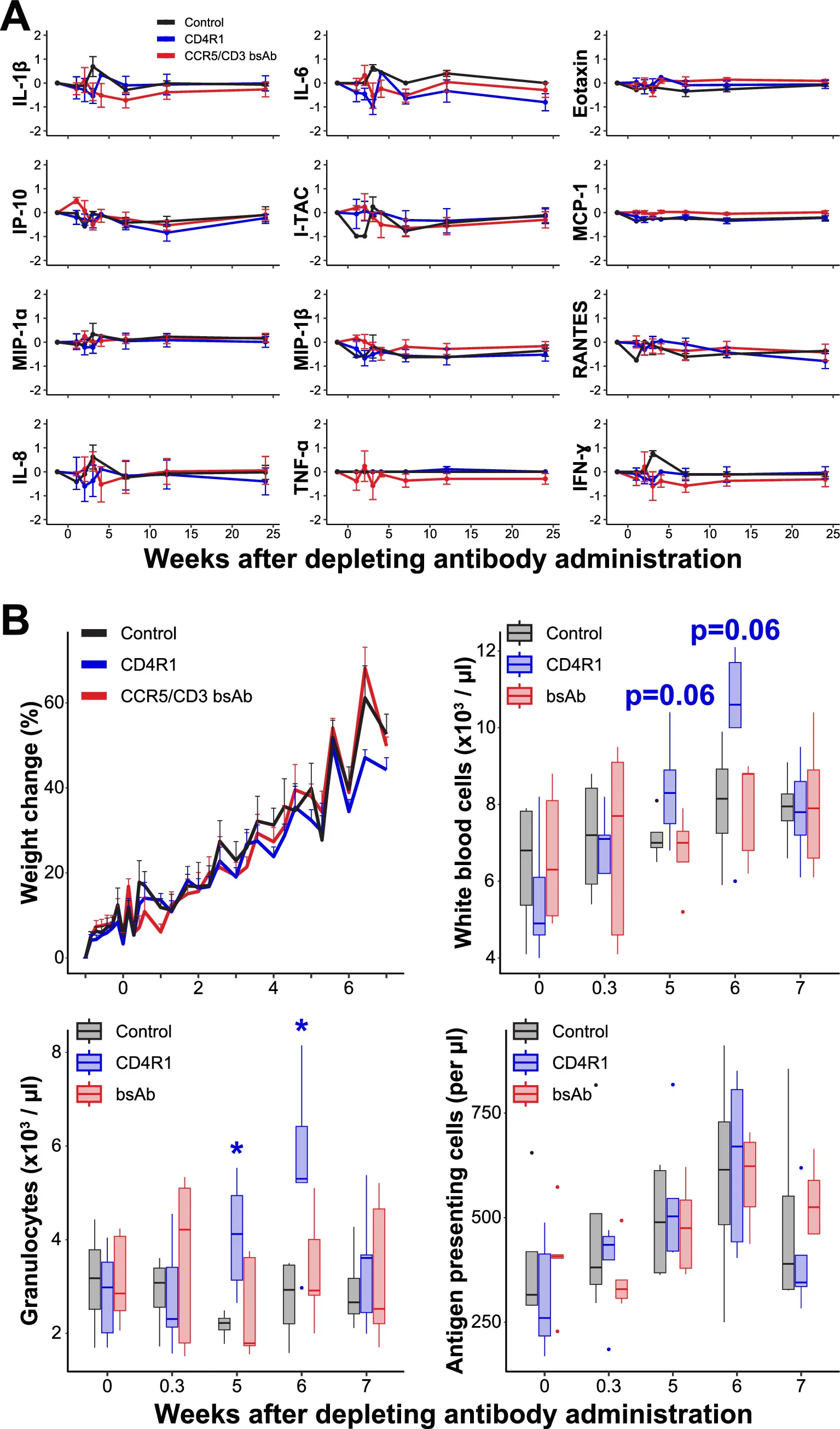
Minimal effects of CD4 or CCR5 depletion on cytokines in blood or major circulating cell subsets. **(A)** Change in the log concentrations of 12 cytokines measured by Luminex are shown. Group averages are plotted with error bars indicating the standard error (n = 4, 5, and 2 for CD4R1, CCR5/CD3 bsAb, and control groups, respectively). There are no significant differences in cytokine concentration that are attributable to depletion using either CD4R1 (administered days 0 and 14 on the x axis) or CD3/CCR5 bsAb (day 0 only). **(B)** Weight changes (upper left) and counts of white blood cells, granulocytes, or antigen-presenting cells (CD3^−^CD20^−^HLA-DR^+^) after depletion. Data are presented as mean values +/- SEM (n = 9, 8, and 7 for CD4R1, CCR5/CD3 bsAb, and control groups). There are no significant between-group differences in weight. A significant elevation in granulocyte counts is observed in CD4R1-recipient animals 5-6 weeks after depletion (middle graph). Boxplots display the median value, lower hinge at the first quartile, upper hinge at the third quartile, lower whisker at the smallest value no further than 1.5 times the inter-quartile range (IQR) from the lower hinge, and upper whisker at the largest value no further than 1.5 times the IQR from the upper hinge. Asterisks indicate p = 0.01 for comparison of granulocyte counts in the CD4R1 group vs. the other two groups, by Wilcoxon testing (two-sided; n = 5 per intervention group and 4 in the control group). Source data

In group C, relative depletion of circulating CCR5^+^ cells persisted in some treated animals for ≥34 weeks following a single administration of bsAb, while one macaque saw restored CCR5 expression by the same time point. It was notable that the T cell count recovered quickly, by ~4 weeks after bsAb infusion, while CCR5 depletion persisted for a long period. This pattern could be explained by transient T cell margination due to CD3 engagement combined with the intended immune-mediated destruction of CCR5^+^ cells. We hypothesized that the idiopathic CD3^+^ lymphopenia might be caused or accompanied by immune activation; however, we did not detect any statistically significant differences in inflammatory cytokine production between bsAb-treated animals and controls by multiplex Luminex assay (Extended Data Fig. 3a; depletion of cells from tissues is addressed in ref. ^31^). Therefore, the RCVR-depleting agents tested here had surprisingly long-lasting effects on blood and tissue immunophenotypes but, importantly, bsAb treatment did not result in acute inflammatory cytokine production or persistent lymphopenia.

Animals in both groups appeared to tolerate the depletion procedure. Some macaques in each group experienced mild hypersensitivity-type reactions, which are not uncommon in macaques receiving intravenous antibody and resolved within hours. Body weights increased comparably in control and depletion groups (Extended Data Fig. 3b). At 5–6 weeks after infection, anti-CD4-recipient animals had more circulating white blood cells and granulocytes than control or bsAb-recipient animals (Extended Data Fig. 3b). This mild granulocytosis in group B was not accompanied by any clinical signs.

## Viral rebound was significantly delayed in CCR5-depleted macaques

**Fig. 3:**
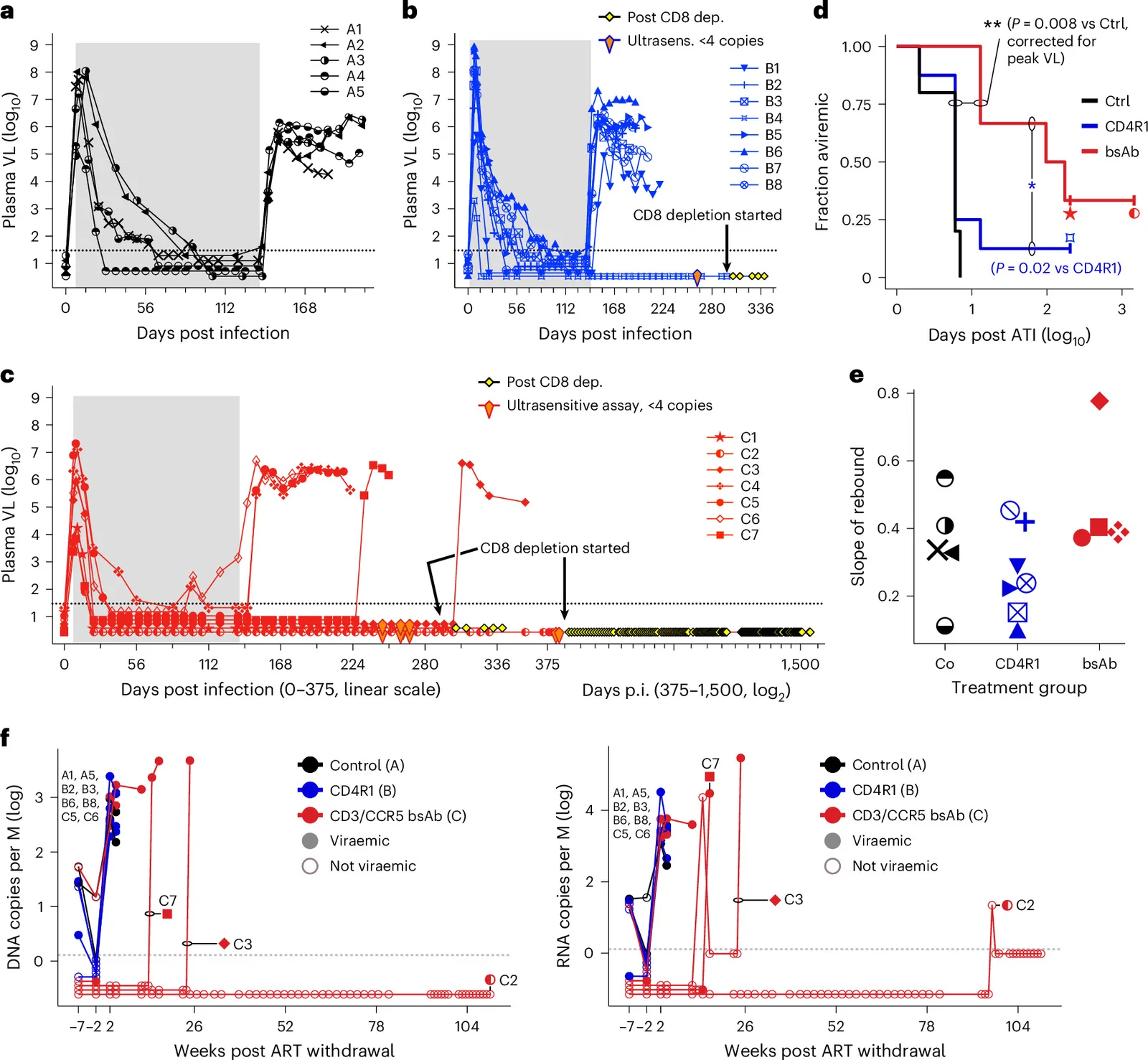
Virologic outcomes of depletion following treatment interruption. **a**–**c**, Grey shading indicates the period of ART treatment. The dotted line represents the limit of detection of our viral-load assay (30 copies per ml or lower); elongated kite shapes show occasions on which high-sensitivity assays were performed, for which the limit of detection was 4 copies per ml. **d**, Survival curves show the fraction of fully suppressed macaques in each group that maintained aviraemia versus time after treatment interruption. Time-to-rebound analysis was conducted using a Cox proportional hazards model, incorporating treatment group and log_10_ peak viral load during acute infection as covariates. Significance was determined using two-sided Wald test without adjustment for multiple comparisons. **e**, The slope of viraemia rebound in each animal was calculated by linear regression of increasing viral loads (that is, before the first post-rebound decline in viral load and including the peak value) occurring after treatment interruption. Additional explanation of the calculation is given in Methods. **f**, Cell-associated viral DNA (left) or RNA (right) copies per million PBMC. Filled markers indicate that the animal had detectable viraemia at the nearest tested time point; open markers indicate that the animal did not have detectable viraemia. All samples plotted below the dotted line had no detectable nucleic acid. Source data

ART was withdrawn after 18 weeks of therapy to assess viral rebound, defined as reappearance of >29 copies of SIV per ml of plasma in an animal that had exhibited complete suppression at the time of ART interruption. Peripheral viraemia was observed within the first 7 days in all controls (Fig. 3a) and 6/8 CD4R1-treated animals (Fig. 3b), while the first bsAb-treated animals did not rebound until 13 days (2/6; Fig. 3c). The other 4 bsAb recipients delayed rebound until 97 or 173 days, or did not rebound before the end of the experiment (29 weeks in one case and 4 years in the other). In every instance of rebound, the viral load reached a set point greater than 10^4^ copies per ml, with no animals exhibiting post-rebound control.

Cox regression using a model that included peak viral loads and treatment group as predictors showed that lower peak viral load and CD3/CCR5 bsAb treatment were each associated with significant delays in rebound (*P* = 0.001 and *P* = 0.008, respectively; Fig. 3d). Thus, limited initial viral replication was probably required to provide the opportunity for delayed rebound induced by the bsAb. The model estimated a 70% reduction in rebound hazard associated with each log-unit decline in peak viral load and a 95% reduction in hazard due to bsAb treatment. Inheritance of a beneficial MHC allele was not associated with delayed rebound (*P* = 0.32). Across treatment groups, 8 of 14 macaques rebounding within 2 weeks of ART cessation expressed Mamu-A*01, -B*08 or -B*17 (57%; see Extended Data Table 1), while only 2 of 5 later-rebounding macaques did so (40%). (Of the 3 animals that never rebounded, only 1 was A*01+; this bsAb recipient was necropsied 204 days after ART withdrawal.) There was no apparent difference between groups A, B and C in the slope of increase in viraemia when rebound did occur (*P* = 0.56; Fig. 3e).

**Extended Data Fig. 4:**
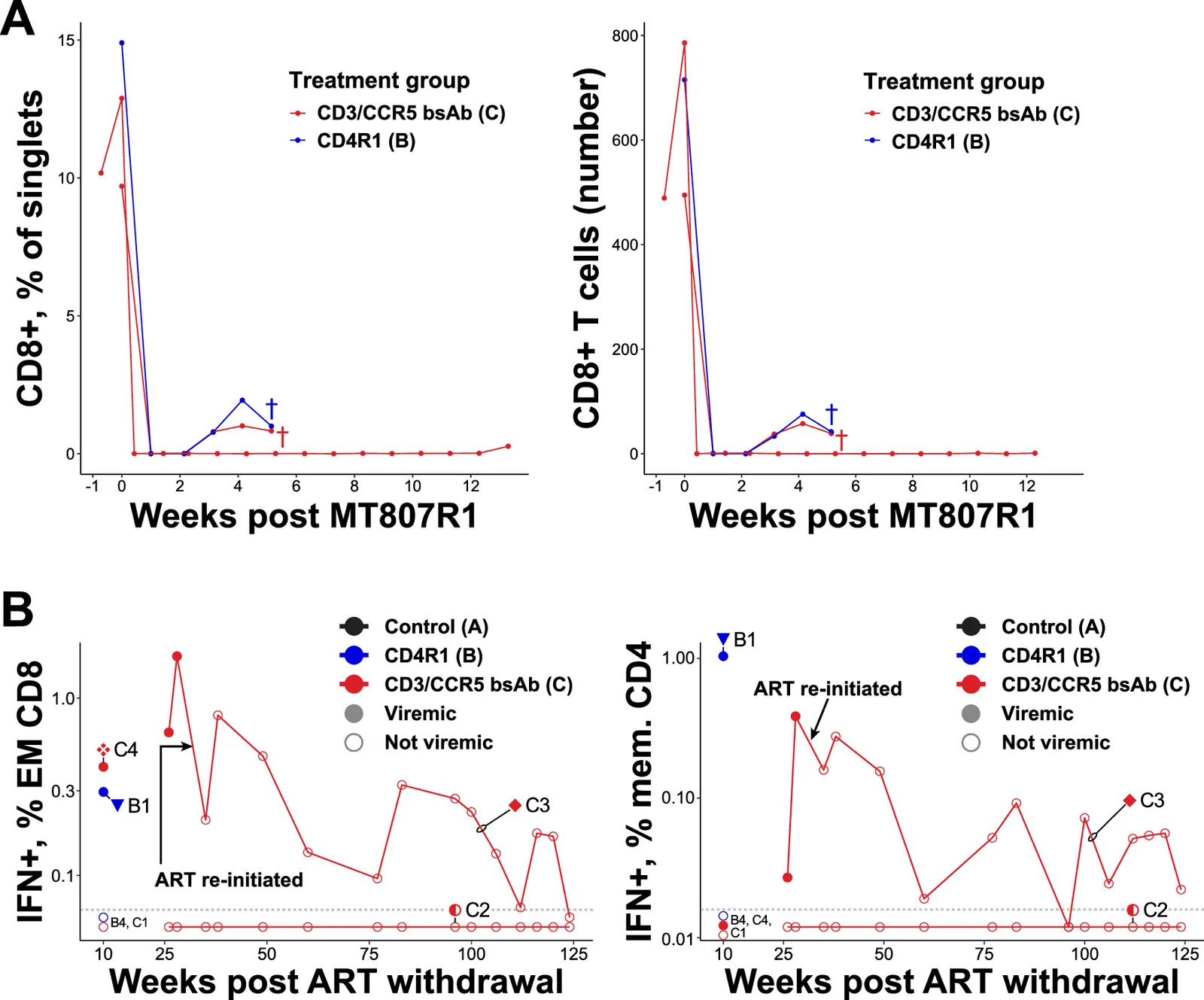
Remission is not caused by T-cell control. **(A)** Depletion of CD8^+^ T cells from macaques treated with CD4R1 or CD3/CCR5 bsAb that were exhibiting post-treatment control. One CD4R1 recipient (B4) and two bsAb recipients (C1 and C2) were treated. The rhesus IgG1 recombinant anti-CD8 alpha (MT807R1) monoclonal antibody was engineered and produced by the Nonhuman Primate Reagent Resource (NIH Nonhuman Primate Reagent Resource [NHPRR] Cat. #PR-0817, RRID:AB_2716320). The antibody was administered in four doses on days 0, 3, 7, and 10 according to NHPRR instructions. The crosses show when two animals were necropsied to allow testing of tissue for residual virus (Fig. 4). **(B)** Immune-response assays were periodically performed using the limited cells available from these newborn macaques. Shown are IFN-γ production by CD3^+^CD8^+^CD95^+^CCR7^−^ PBMC (left) or CD3^+^CD4^+^CD95^+^ PBMC (right) after stimulation with overlapping 15-mer peptides from the SIV gag gene, assessed by cytokine flow cytometry. The figure shows results from one control macaque, two group-B (CD4R1) macaques, and five group-C (bsAb) macaques. Two of the latter animals (bsAb recipients) were tested multiple times during extended follow up. ART was re-initiated in one macaque so that the animal could continue serving as a cage-mate to an animal that remained in remission. Filled markers indicate that the animal had detectable viremia at the nearest tested time point; open markers indicate that the animal did not have detectable viremia. Source data

We next wished to determine the extent of viral clearance, as defined by the absence of detectable viraemia and failure to detect replication-competent virus in any sample, in the 3 macaques that achieved durable post-ART control (no rebound to the end of the experiment, after >6 months). The single CD4R1-treated and 2 bsAb-treated animals in remission were first tested for residual viraemia. No virus was detected using a high-sensitivity quantitative PCR (qPCR) assay having a limit of detection of 4 copies per ml (orange kites in Fig. 3b,c). Second, to provoke outgrowth of any replication-competent virus residing in RCVRs, cytotoxic T lymphocytes in all 3 animals were depleted using the rhesusized anti-CD8ɑ monoclonal antibody, MT807R1. Post-treatment flow cytometric analysis demonstrated complete depletion of blood CD8^+^ T cells in all animals (Extended Data Fig. 4a). Despite this transient elimination of the cytotoxic T lymphocyte response, rebound did not occur, suggesting that CD8^+^ T cells were not the primary mechanism of control (black/yellow diamonds in Fig. 3b,c). Indeed, T cell responses to SIV were associated with plasma viraemia and generally not measurable in control animals (Extended Data Fig. 4b).

We next evaluated cell-associated SIV nucleic acid in PBMCs before and after treatment interruption in a subset of animals for which sufficient cells were available, including 2 controls, 4 CD4R1 recipients and 5 bsAb recipients. Cell-associated DNA declined between weeks 12 and 17 after infection (weeks −7 and −2 relative to ART interruption), reflecting continued effective suppression by ART (Fig. 3f). After ART interruption, detection of cell-associated SIV DNA was strongly associated with plasma viraemia: animals experiencing rapid rebound also manifested increasing cell-associated SIV DNA. However, SIV DNA was not detectable in bsAb-recipient animals with delayed rebound until they became viraemic (Fig. 3f). In a bsAb recipient followed for >4 years of remission, cell-associated SIV DNA was never detected after ART withdrawal.

Cell-associated SIV RNA (Fig. 3f) demonstrated similar dynamics, with a decline between 12 and 17 weeks after ART initiation, and rapid rebounders manifesting RNA very soon after ART withdrawal. bsAb recipients with delayed rebound were also delayed in RNA production. Interestingly, in one bsAb recipient, measurable PBMC-associated viral RNA preceded detectable viraemia by many weeks. Finally, in the bsAb-recipient macaque that remained aviraemic for 4 years, one instance of possible RNA detection (2 copies in 91,304 cells), without DNA or viraemia, occurred at week 97. Although contamination could not conclusively be ruled out, the amplification kinetic appeared correct and the PCR product had the expected sequence.

In sum, these data provide remarkable evidence that CD4 or CCR5 depletion in recently infected infants can alter both the kinetics of SIV spread, as reflected in viraemia, and RCVR establishment, such that 1/8 CD4R1-treated and 4/7 bsAb-treated animals achieved remission that was maintained for >3 months.

## RCVR depletion achieved by bispecific antibody

**Fig. 4:**
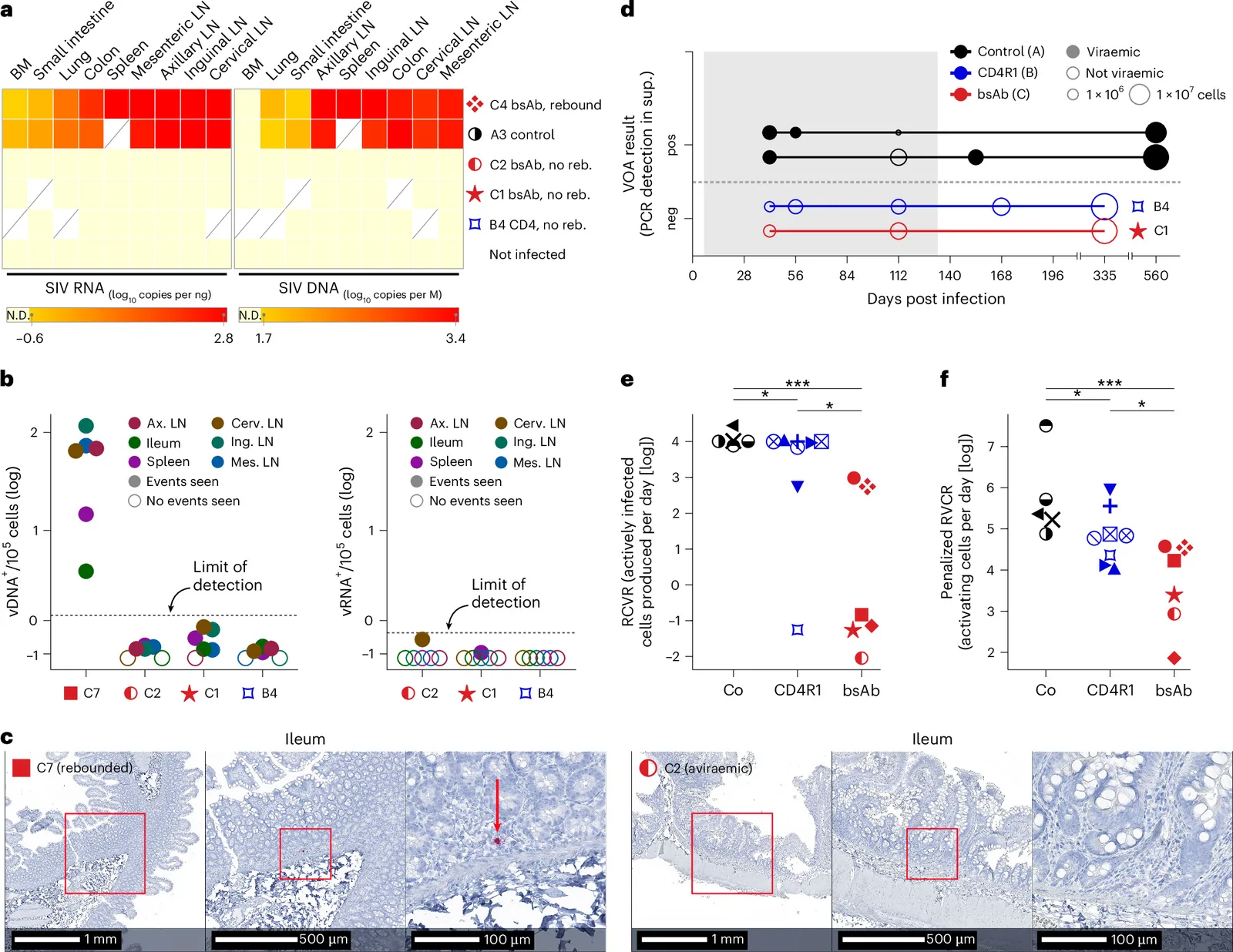
Tests for residual virus in tissue samples. **a**, Quantitative PCR results from the tissues of 2 bsAb-treated and 1 CD4R1-treated animal that did not rebound during the period of follow-up (C2, C1, B4; rows 3–5; ‘no reb.’), along with results from positive and negative controls. Viral nucleic acid was detected in all tissues of a necropsied control animal (second row) or a necropsied bsAb recipient that did rebound (first row), but not in any tissues of the non-rebounding bsAb and CD4R1 recipients. For each tissue, the cDNA from 300 ng of total RNA (left) or DNA from ~2.4 × 10^5^ cells (right) were analysed. **b**, Quantified DNAscope (left) and RNAscope (right) analyses of viral nucleic acid in bsAb- or CD4R1-treated macaques. The limits of detection show the statistical upper limit of nucleic acid detected by the assays in a large number of uninfected control tissues. The RNAscope results for the positive-control tissues are not plotted due to high numbers of virions detected around vRNA^+^ cells or deposited on follicular dendritic cells, which would make a precise count very labour intensive. **c**, Representative staining by RNAscope of viral nucleic acid in ileum from two animals in group C, demonstrating the high quality of the imaging. For animals B4, C1 and C2, ~10–60 high-power fields containing a total of 125,000–750,000 nuclei were examined for each tissue. **d**, Viral outgrowth was detected from lymphocytes of control macaques when the animals were viraemic (filled markers) or aviraemic (open markers). No outgrowth was detected from 8 lymphocyte samples from depleted macaques that never rebounded. The number of cells used for each assay is indicated by the size of the marker. VOA, viral outgrowth assay. **e**, Estimation of the size of the viral reservoir before interruption of ART. Between-group differences were tested using a generalized linear model (GLM) with a Gamma distribution and log link, controlling for both protective MHC class I alleles and peak viral load. Significance was assessed using two-sided Wald *t*-test on the model coefficients, without adjustment for multiple comparisons (*P_AB_ = 0.0241, *P_BC_ = 0.0316, ***P = 0.0007). **f**, Moderation of the estimate in viral reservoir size by adjusting for peak virus load: RCVR estimates were inflated for animals with lower peak viraemia. Statistical differences were tested using the same approach except that the presence of protective alleles was the only covariate (*P_AB_ = 0.0117, *P_BC_ = 0.0200, ***P = 0.0001). Source data

Clinical experience and mathematical modelling have shown that indefinite control over viraemia is an attractive but problematic experimental endpoint, because the RCVR can be undetectable and still cause rebound after prolonged aviraemia^38–40^. Three macaques (B4 [female], C1 [male] and C2 [male]) appeared to have been potentially cured in our study, with no evidence for continued infection after extended follow-up, including after CD8^+^ T cell depletion. We chose to follow one of these animals long term (C2, a bsAb recipient) and to necropsy the other two (B4 and C1) to rigorously search for persisting virus. Bone marrow, intestine (duodenum and colon), lung, spleen and multiple lymph nodes (cervical, axillary, inguinal, mesenteric) were collected for qPCR and RNA/DNAscope in situ hybridization (ISH). In contrast to bsAb-treated animals with rebound following ART interruption and to an ART-alone control, no residual SIV RNA or DNA was detected by qPCR in any of the assayed tissues (Fig. 4a). Similarly, DNAscope and RNAscope analysis revealed no viral nucleic acid in the tissues of the aviraemic macaques, while an identical survey of a viraemic, bsAb-treated animal revealed unambiguous nucleic acid detection in 6/6 tissues assessed (Fig. 4b,c). Viral outgrowth assays from lymph-node cells, sampled over time and at necropsy, failed to recover SIV in 8 of 8 attempts from the two non-rebounding and necropsied animals (4.7 × 10^7^ or 3.4 × 10^7^ total cells tested; Fig. 4d), while lymph-node biopsies from 2 control animals did grow SIV both when the animals were viraemic and when they were not. Thus, treatment with CD4R1 and CD3/CCR5 bsAb impacted the SIV reservoir both in blood (Fig. 1d,e) and in tissues (Fig. 4).

Finally, to gain quantitative insight into the reduction in the RCVR resulting from CD4R1 or CCR5/CD3 bsAb treatment, we used the survival curves for rebound viraemia and the statistical tools in refs. ^41,42^. These tools integrate viral load and viral outgrowth data to provide information about the likely size of the underlying RCVR. Using this model to calculate most-likely RCVR sizes for each macaque (measured in terms of the rate at which the RCVR produces actively infected cells), we found that the estimated RCVR in both CD4R1 and bsAb recipients was smaller than that in control animals (*P* = 0.02 and *P* = 7 × 10^−4^, respectively; Fig. 4e) in a model that corrected for both peak viral loads and inheritance of a beneficial MHC allele. The model also found that higher peak viral loads were significantly associated with a larger RCVR at ART interruption (*P* = 4 × 10^−6^), while controller alleles had no association. Interestingly, the regression model estimates that a one log-unit increase in peak viral load is associated with a 6.6× increase in RCVR, CD4R1 treatment with a 13× decrease in RCVR and bsAb treatment with a 152× decrease. If these estimates are approximately correct, then CD4R1 treatment produced an effect that is similar to a 23-fold reduction in peak viraemia and bsAb treatment an effect similar to a 460-fold reduction in peak viraemia.

We further explored the effect of peak viraemia on the RCVR using a penalization procedure instead of including peak viral load as a covariate in the model: calculated RCVRs at ART interruption were inflated for animals manifesting lower peak viraemia. Thus, the RCVR estimate for the single animal with highest peak viraemia was not adjusted, while all others were increased by the number of log units corresponding with their peak viraemia decrement. (Since the full regression model estimates that a 10× increase in peak viraemia is associated with only a 6.6× increase in RCVR, this penalization procedure is an overcorrection.) In this penalized analysis, which also corrected for inheritance of a beneficial MHC allele, the estimated RCVR after treatment with CD4R1 or bsAb was significantly smaller than that in control animals (*P* = 0.01 and *P* = 10^−4^, respectively; Fig. 4f). This regression estimated that CD4R1 treatment was associated with a 20× decrease in RCVR and bsAb treatment with a 219× decrease.

## Discussion

We found that newborn macaques receiving depletion therapy can achieve durable remission, including aviraemia maintained for up to 4 years following treatment interruption and despite depletion of CD8^+^ T cells. This hopeful finding is perhaps surprising. Mathematical modelling suggests that a 2,000-fold reduction in the reservoir is required to delay rebound by 1 year in most people^41,42^, while a 5,000-fold reduction is required to sustain remission for 3 years. An efficiency of depletion of even 99.9% in peripheral blood and less in tissues^31^ might seem unequal to the task. However, CCR5 depletion probably alters key parameters that control viral rebound, such as the rate at which reactivated virions encounter newly infectable targets, or the likelihood of reactivation from latency. Indeed, as little as 55–90% CCR5 knockout via gene editing suffices to help xenograft mice resist HIV infection^30^. Complete elimination of all latently infected cells may also be unnecessary because under some circumstances, it appears that host defences can eliminate nascent infectious foci^43,44^.

Most importantly, CCR5 depletion surely alters the rate at which virus from reactivated cells can find new targets—known to be a key parameter controlling rebound^41^. Thus, the mechanism of remission in our study may involve not only elimination of infected cells, but also depletion of cells that are vulnerable to infection by reactivated SIV. Reactivated virus finds no foothold among remaining immune cells, while the RCVR is gradually diminished due to toxicity of viral replication and/or attrition of the cells with time. The reported ‘Chicago patient’, for example, received a CCR5Δ32/Δ32 stem-cell transplant and rebounded to 780 copies per ml of R5-tropic virus after a first analytic treatment interruption, but after an additional 22 months of treatment was able to achieve >10 months’ ART-free remission^45^. ART was therefore potentially curative in this individual with a persistent HIV reservoir and few infectable target cells, whereas ART alone is virtually never curative otherwise.

A previous study testing one administration of CD4R1 antibody much later in infection^28^ demonstrated a reduction in cell-associated SIV DNA in PBMCs, as well reduced viral outgrowth for animals that started ART treatment earlier (2 months vs 10 months post infection). Another group of investigators, administering CD4R1 31 weeks after infection to macaques that had received early ART, demonstrated a non-significant trend to delayed rebound after ART withdrawal^29^. In both cases, the CD4 depletion achieved in rectum averaged ~50%. These studies and our results seem to agree that broad depletion of SIV target cells can achieve a measure of reservoir depletion, while differing in the timing of antibody administration and consequently in the size of the targeted reservoir. Depletion of CD4^+^ T cells before SIV infection was found in two studies to cause inability to control SIV, including set-point viral loads 100× higher than in controls^46,47^. The authors concluded that antiviral CD4^+^ T cells help limit SIV replication, which is an additional possible explanation for better performance of the CCR5-targeting strategy versus the CD4-targeting strategy in our study. Furthermore, surface CD4 and CD3 expression are often downregulated acutely following HIV/SIV infection^48–50^, which may limit the effectiveness of CD4-targeting strategies.

Although CCR5 is needed for infection by most HIV and SIV variants, it has not been seen as a strong marker for the reservoir in later infection. The reservoir under ART is concentrated among central memory (T_CM_) and transitional memory (T_TM_) CD4^+^ T cells^51^. A less differentiated subset of long-lived cells with high self-renewal capacity, the stem-cell memory CD4^+^ T cell (T_SCM_), has been identified as a contributor to long-term HIV persistence^52,53^. The persistent reservoir has also been described according to T cell function and homing capacity: Th17 and Th1/Th17 CD4^+^ T cells, as well as cells expressing CCR6 and CXCR3, show increasing contribution to the reservoir with duration of ART^54,55^. Chomont and colleagues found that CD4^+^ T cells expressing PD‑1, TIGIT and LAG-3 alone or in combination are enriched for persistent HIV during ART^56^.

Notably, however, this field-wide examination of the HIV reservoir has predominantly focused on chronically infected individuals. CCR5 is probably a good marker of the RCVR in early infection because CCR5^+^ cells are the first to be infected^57–59^ even though some of these CCR5^+^ cells, including CCR5^+^ pre-Tfh, can cease expression in the process of differentiation to longer-lived cells that contribute to the later RCVR^60^. It is additionally possible that CCR5/CD3 bsAb serves as an entry inhibitor by preventing access of the virus to its co-receptor. If so, then blockade of CCR5 with binding antibodies and/or small molecules may raise the chance of post-treatment cure. It has been suggested that there is a window of vulnerability following allogeneic haematopoietic stem-cell transplantation, during which T cell activation and increased CCR5 expression coincide with virus reactivation^61^. If CCR5 expression is crucial for re-establishing the reservoir during such a window, then CCR5 depletion could be beneficial.

The extent and durability of CCR5 depletion achieved in this study with a single shot were unexpected. CCR5 expression has been associated with T cell activation^62–65^, suggesting that CCR5 expression is dynamic and therefore unlikely to be susceptible to long-term depletion with a single dose. An early detailed study of CXCR4 and CCR5 regulation in response to activation of human T cells, however, showed that CCR5 expression was unchanged throughout 12 days of continuous stimulation with PHA or anti-CD3 (ref. ^66^). A detailed review described unexpectedly broad CCR5 expression across many human CD4^+^ T cell subsets including Th1, Th17 and T-reg cells and on CD8^+^ T cells expressing Granzyme K^48^. In both humans and macaques, some PD-1^high^CXCR5^+^ Tfh express CCR5, which could be key to the success of anti-CD3/CCR5 in RCVR depletion^60,67^. It is clear that the presence of CCR5 on the cell surface can be reduced by receptor internalization due to ligand binding^68^. Nonetheless, the durability of the depletion we observed suggests that CCR5 expression is primarily a stable characteristic of certain cell lineages.

Adults with the CCR5 ∆32/∆32 homozygous genotype have not shown any obvious immunodeficiency, apart from reports of increased susceptibility to symptomatic West Nile virus encephalitis^69–71^. This apparent tolerance of lifelong CCR5 deficiency suggests that transient depletion of CCR5-expressing cells to achieve HIV cure may be an acceptable risk. Nevertheless, in its current form, the CCR5/CD3 bsAb treatment raises concerns related to broad depletion of both HIV-infectable and -uninfectable cells, including CCR5^+^CD8^+^ T cells, suggesting the importance of testing for any increased susceptibility to opportunistic infection, as well as any effect on the ability to control HIV viraemia when the infection cannot be cured^72^.

Our study demonstrates that targeting CCR5-expressing cells using the host immune system may be a surprisingly effective route to HIV cure, at least for infants that can be treated soon after infection. The impact of CCR5/CD3 bsAb on adult SIV infection may differ due to different viral dynamics and host immune function. Even when this approach alone fails to eradicate the virus, however, the apparent reservoir reduction and target-cell depletion achieved could enhance the efficacy of other immunotherapies in development. For example, exciting work has recently shown that vectored delivery of HIV bnAbs can provide durable ‘functional cure’ for SHIV-infected infant macaques^27^; however, the latent reservoir remains intact and the animals can become viraemic after depletion of circulating bnAbs or CD8^+^ T cells^73^. Anti-CD3/CCR5 bsAb might prove to be part of an effective integrated approach to depletion of both active and latent reservoirs, offering the promise of complete viral clearance to certain PWH harbouring limited latent HIV.

## Methods

### Experimental animals and samples

This study was approved in advance by the University of California, Davis (UC Davis) Institutional Animal Care and Use Committee (IACUC; approval number 19134) and was performed at the National Biomedical Research Institute (NBRI). Housing, medical care and all procedures were performed in accordance with UC Davis IACUC-established policies. UC Davis has an Animal Welfare Assurance on file with the NIH Office of Laboratory Animal Welfare and is fully accredited by the Association for the Assessment and Accreditation of Laboratory Animal Care International. Twenty-five newborn^74^ rhesus macaques (*Macaca mulatta*) of Indian origin were selected from the conventional colony at NBRI.

The animals were randomized to experimental groups before SIV inoculation. The sex ratios did not differ significantly by group either when all newborns were considered (*P* = 0.54 by Fisher’s exact test; Extended Data Table 1) or when only those successfully infected were considered (*P* = 0.17). All animals tested negative for HIV-2, SIV, simian T cell lymphotropic virus type 1 (STLV) and type-D retrovirus at the start of the study. The volume of whole blood collected from newborns and infants each month was limited to a total of <8 ml kg^−1^.

Eleven juvenile rhesus macaques were selected for the experiment shown in Extended Data Fig. 1. The animal treated with bispecific antibody was female and inherited Mamu-B*08. The 10 controls included 4 males and 6 females, all Mamu-B*08 negative. All 11 macaques were Mamu-A*01 negative and Mamu-B*17 negative.

Animals were housed at the NBRI. Animals were administered ketamine-HCl intramuscularly at 10 mg kg^−1^ body weight when necessary for immobilization. Analgesics were administered at the discretion of the NBRI veterinary staff to minimize pain and discomfort. Plasma and PBMCs were prepared from EDTA-anticoagulated blood and stored at −80 °C as previously described^75^. Mononuclear cells from lymph node biopsies were also prepared and stored as previously described^75^.

### Virus and cART

Macaques were challenged orally with 50,000 TCID_50_ of SIVmac251 twice on the day of inoculation (15–23 days of age) as previously described^76^. Challenge virus was provided by the NBRI Virology Core. Antiretroviral triple therapy, consisting of tenofovir disoproxil fumarate (5.1 mg kg^−1^), emtricitabine (40 mg kg^−1^) and dolutegravir (2.5 mg kg^−1^), was delivered subcutaneously each day^77^. The slope of viral rebound following ART withdrawal (Fig. 3e) was calculated in log units per day using R’s ‘lm’ function in the base package, ‘stats’. For each animal, the monotonically increasing values after ART withdrawal were analysed in a regression model using log_10_ viral load as a dependent variable and days after regression as predictor. The slope of the resulting model for each animal gives the slope of rebound plotted in Fig. 3e.

### Cytotoxic antibodies

Anti-CD3/CCR5 bispecific antibody, produced and purified as previously described^31^, was infused intravenously at 3 mg kg^−1^. Rhesus IgG1 recombinant anti-CD4 [CD4R1] antibody was acquired from the NIH Non-human Primate Reagent Resource (NHPRR) and administered at 50 mg kg^−1^ by intravenous infusion according to manufacturer instructions. Animals were pretreated with diphenhydramine to reduce the risk of anaphylactoid reactions to the antibody therapy. Oxygen was administered post infusion at the veterinarian’s discretion.

### Immunophenotyping and whole-blood staining

PBMC and lymph node cells were stained and analysed by flow cytometry as previously described^31^. At least 0.5 M cells were used for immunophenotyping assays. Absolute cell subset counts were obtained with dual-platform techniques utilizing flow cytometry to provide the percentage of a given cell subset among total blood leucocytes along with a haematology analyser. EDTA-anticoagulated whole blood was assessed with an ABX Pentra 60C+ haematology analyser (HORIBA) or stained with monoclonal antibodies against cell markers including CD3 (clone SP34-2), CD4 (clone L200), CD8 (clone SK1), CD11c (clone N418), CD11b (clone ICRF44), CD14 (clone M5E2), CD16 (clone 3G8), CD20 (clone 2H7), CD25 (clone M-A251), CD28 (clone CD28.2), CD80 (L307.4), CD95 (clone DX2), CD159 (NKG2A, clone REA110), CD185 (CXCR5, clone SPRCL5), CD195 (CCR5, clone 3A9), CD279 (PD-1, clone EH12.2H7) and HLA-DR (clone G46-6). The anti-CCR5 clone, 3A9, and the clone used for bsAb construction (5C7) were shown to bind simultaneously to CCR5-expressing cells (Extended Data Fig. 6). All fluorochrome-conjugated monoclonal antibodies were purchased from BD Biosciences, BioLegend, Thermo Fisher or Miltenyi Biotec. Lysis and fixation of whole-blood samples were performed using the TQ-Prep Workstation and IMMUNOPREP reagent system (Beckman Coulter). Cells were acquired on a BD FACSymphony A3 Cell Analyzer (BD Biosciences) and data were analysed using FlowJo software (BD Biosciences).

### Cytokine flow cytometry assay

Levels of SIV-specific cytokine-producing cells were determined by flow cytometry after stimulation with sequential 15-mer peptides (overlapping by 11 amino acids) comprising the SIVmac239 Gag, Rev or Nef (provided by NIH AIDS Reagent Program) and anti-CD28 (clone CD28.2, NIH NHPRR) and anti-CD49d (clone L25, BD Biosciences) monoclonal antibodies. Cells were incubated with peptides (or DMSO control) and co-stimulatory antibodies for 1 h, followed by addition of Brefeldin A (Sigma-Aldrich) for an additional 8 h. Cells were stained for surface markers with LIVE/DEAD Fixable Aqua Dead Cell Stain kit (Thermo Fisher), anti-CD3-APC-Cy7, anti-CD4-BV650, anti-CD8-PerCP-Cy5.5, anti-CD95-APC and anti-CCR7-BV421 (clone 3D12). Cells were then washed, permeabilized using a Cytofix/Cytoperm solution kit (BD Biosciences), and intracellularly stained with anti-TNF-Alexa 700 (alone MAB11) and anti-IFN-γ-PE-Cy7 (clone B27). After staining, cells were washed, fixed with 1% paraformaldehyde in staining buffer (PBS containing 1% FBS) and acquired using Fortessa or FACSymphony cytometers operated by FACSDiva software (BD Biosciences).

### CD8 T cell depletion

Rhesusized anti-CD8 alpha antibody [MT807R1]^78,79^ acquired from the NHPRR was used to deplete CD8^+^ lymphocytes. The antibody was administered according to manufacturer instructions (SOP A06-01): at 10 mg kg^−1^ subcutaneously on day 0 and at 5 mg kg^−1^ intravenously on days 3, 7 and 10 of the treatment protocol.

### Viral detection assays

SIV RNA in plasma was measured by RT–qPCR as described^17,80^. The plasma volume used for viral load measurements was 0.35 ml or 0.7 ml depending on the amount of sample available. Key tissues collected at necropsy were assessed for viral RNA and DNA. Tissue samples were stored in RNA Later (Invitrogen) at −20 °C until purification and quantitative PCR analysis. RNA and DNA were purified using a Qiagen All Prep kit according to manufacturer instructions and stored at −20 °C. For RNA, cDNA was generated as previously described^81^. The cDNA and purified viral DNA were used for qPCR using QuantiTect probe PCR master mix (Qiagen) and optimized concentrations of forward primer (TGTGTGGGAGACCATCAAGC), reverse primer (GCTCCCTAAGTTGTCCTTGTTG′) and probe (FAM/ACGAGGAGGCTGCAGATTGGGACTTGCA/NFQ/MGB-3′) in a QuantStudio 12K Flex real-time cycler (Applied Biosystems). PCR amplicons of the SIVgag target were used to generate a standard curve and line equation for qPCR analysis. For each analysed tissue, (1) 600 ng extracted DNA (from ~2.4 × 10^5^ cells) or (2) the cDNA generated from 300 ng of total RNA were subjected to qPCR. PBMC collected longitudinally were also assessed for viral RNA and DNA. Cells were lysed with 1 ml TRIzol Reagent (Ambion), and RNA and DNA were purified sequentially from half of the lysate using a Direct-zol DNA/RNA Miniprep kit (Zymo) according to manufacturer protocol. Cell-associated SIV RNA/DNA quantification used nucleic acid from a mean of 688,579 cell equivalents based on CCR5 genomic-DNA copies detected. Cell-associated SIV RNA was quantified using a QuantStudio 6 Flex instrument (Applied Biosystems) and a Superscript III Platinum One-Step RT–qPCR System (Thermo Fisher). Primers and reporter probe bind to the LTR and their sequences are GCTAGACTCTCACCAGCACTTG (forward), CTAGGAGAGATGGGAACACACA (reverse) and 56-FAM/TCCACGCTT/ZEN/GCTTGCTTAAAGCCCTC/3IABkFQ (probe). Cycling consisted of 50 °C for 15 min, 95 °C for 2 min, followed by 45 repeats of 95 °C for 15 s and 62 °C for 30 s. To measure SIV DNA, the same reagents were used with *Taq*man Fast Universal PCR Mastermix (Applied Biosystems) and the same cycling except for the 50 °C cDNA synthesis step. The single-copy C-C chemokine receptor type 5 (CCR5) gene was quantified to determine the number of cell equivalents in DNA samples for standardization purposes. Reagents to detect CCR5 are GTCTTCATTACACCTGCAGCTCTCA (forward), AGGATTCCCGAGTAGCAGATGA (reverse) and 56-FAM/AGCAGCGGC/ZEN/AGGACCAGCCCCAAG/3IABkFQ (probe) and cycling was the same as that used to amplify SIV DNA.

### SIV RNA detection in tissues by ISH

The detection of SIV RNA and DNA in multiple tissues was completed using RNAscope, as described^82^. Sections were analysed for the total number of vRNA^+^ and vDNA^+^ per 10^5^ total cells using the HALO software (v.3.0.311.405; Indica Labs) ISH module (v.3.0.3). Sections underwent blinded manual curation to correct false positives.

### Viral outgrowth assays

Mononuclear lymph node cells were mixed with 174xCEM cells (BEI Resources, ARP-272) and activated for 24 h in RPMI 1640 growth media containing fetal bovine serum (FBS; 10%), interleukin-2 (IL-2; 50 U ml^−1^), phytohaemogglutinin (PHA; 5 μg ml^−1^) and ionomycin (1 μg ml^−1^). After 24 h, PHA and ionomycin were removed and cultures were maintained for 28 days or until cytopathic effect was apparent in 174×CEM cells. Cell-free supernatants were collected and stored at −80 °C. Viral RNA was purified from the supernatants using a Qiagen UltraSens virus kit according to manufacturer instructions. cDNA was generated and used for qPCR as described above.

### Estimation of probable RCVR at ART interruption

We applied the formulas and modelled survival curves from refs. ^41,42^ to derive posterior probabilities for the ‘latent reservoir exit rate’ when treatment is stopped, that is, the number of actively infected cells produced per day by the remaining RCVR (expressed in the variable, *A*). Briefly, for macaques that do not rebound to a given time, the probability distribution is given as a normalized product of (1) the likelihood of survival to that time, given possible values of *A* (from the survival curves in ref. ^41^ and http://www.danielrosenbloom.com/reboundtimes) and (2) the prior probability distribution for those values of *A* (uniform or given by results of viral outgrowth assays as in ref. ^83^). For macaques that did rebound, the formula is slightly modified so that the likelihood function for the time of rebound is taken from the slopes of the survival curves at that time. As in ref. ^42^, we then determine a point estimate for each animal by taking the median of the posterior distribution (for macaques that rebounded) or the largest value of *A* for which the likelihood of survival to the follow-up time exceeds 0.5 (for those not rebounding).

### Reporting summary

Further information on research design is available in the Nature Portfolio Reporting Summary linked to this article.

## Supplementary information

Reporting Summary

## Source data

Source Data Fig. 1 Statistical source data.

Source Data Fig. 2 Statistical source data.

Source Data Fig. 3 Statistical source data.

Source Data Fig. 4 Statistical source data.

Source Data Extended Data Fig. 1 Statistical source data.

Source Data Extended Data Fig. 2 Statistical source data.

Source Data Extended Data Fig. 3 Statistical source data.

Source Data Extended Data Fig. 4 Statistical source data.

**Extended Data Fig. 5:**
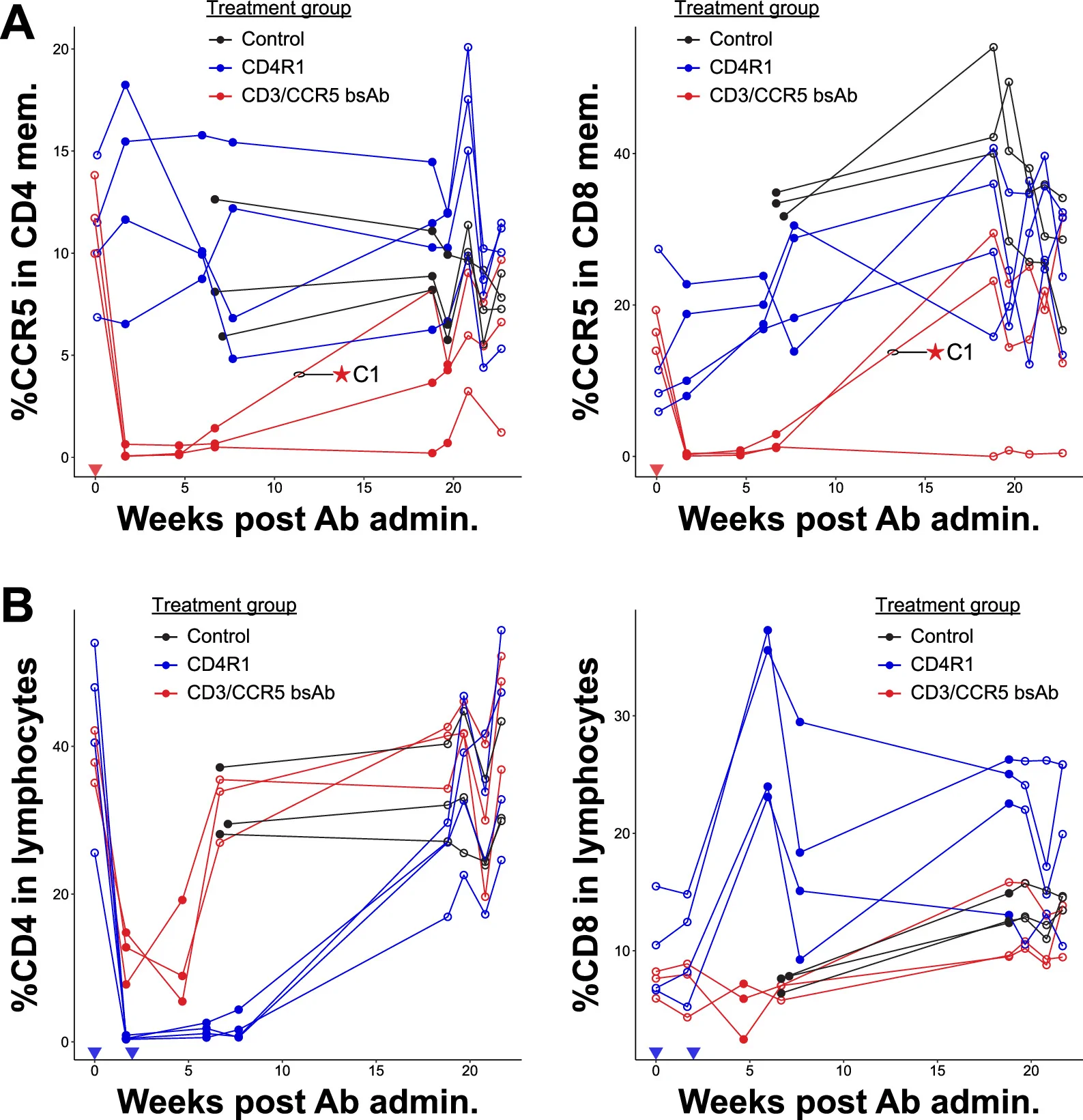
Effects of depleting antibodies on CCR5-, CD4-, and CD8-expressing cells. **(A)** CCR5 expression as a percentage of CD3^+^CD4^+^CD95^+^ memory T cells (left) or as a percentage of CD3^+^CD8^+^CD95^+^ memory T cells (right). Filled markers indicate a significant difference in the percentage of CCR5^+^ cells at that time point in bsAb-recipient vs. control or CD4R1-recipient macaques. **(B)** Fraction among lymphocytes of CD4-expressing (left) or CD8-expressing T cells (right). Filled markers indicate a significant difference in the percentage of CD4^+^ or CD8^+^ cells at that time point in CD4R1-recipient vs. control or bsAb-recipient macaques. These data are plotted separately from Fig. 2 because the blood sampling schedule differed. Absolute-count data are not available. Source data

**Extended Data Fig. 6:**
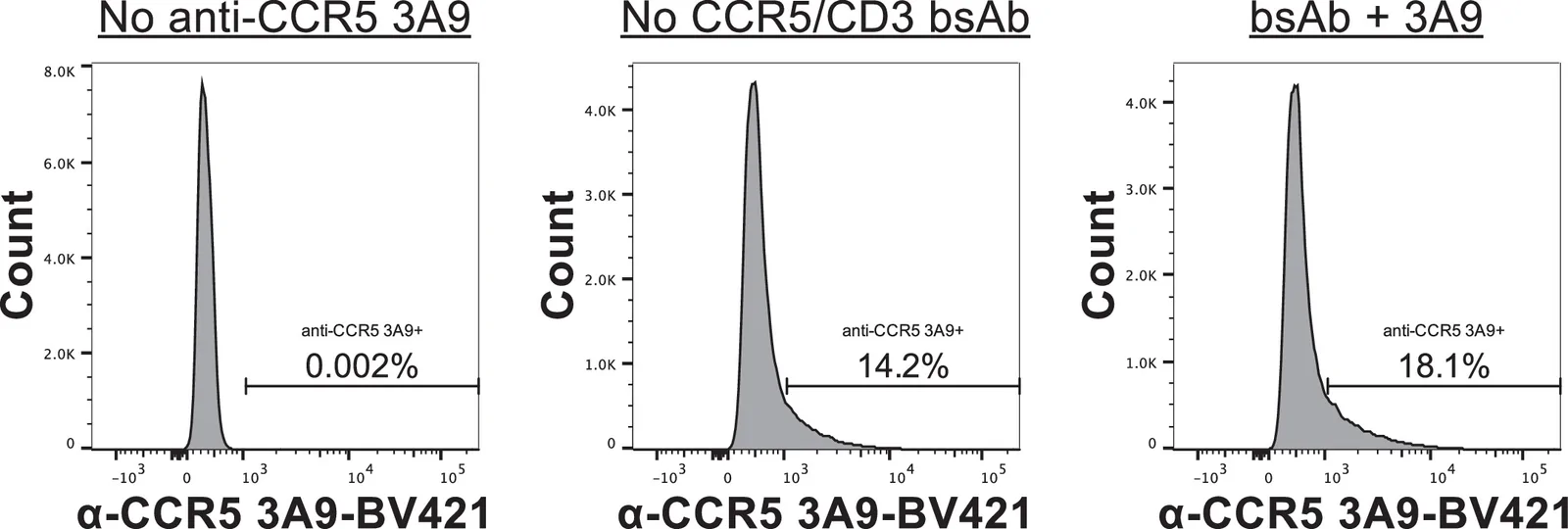
CCR5/CD3 bsAb does not prevent anti-CCR5 clone 3A9 binding. HEK 293 cells were transduced with CCR5 mRNA/LNPs and then stained with fluorescently labeled anti-CCR5 clone 3A9 either in the absence (middle histogram) or presence (right histogram) of CCR5/CD3 bsAb.

Source Data Extended Data Fig. 5 Statistical source data.

## Figures and Tables

**Extended Data Table 1:**
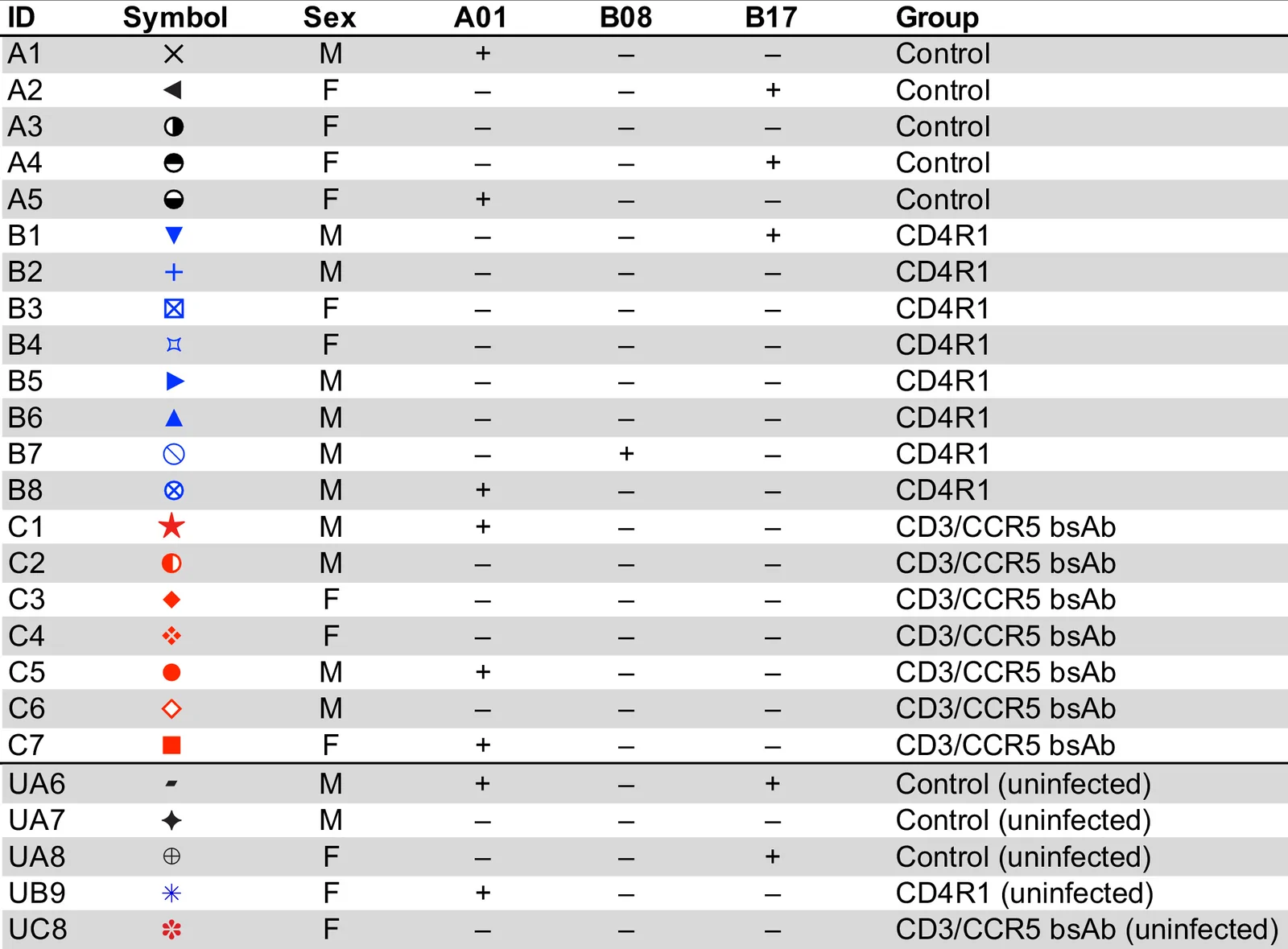
Sex and beneficial MHC alleles of macaques assigned to these studies

## Data Availability

The data that support the findings of this study can be found in the Source data provided with this paper.
