# Supplementary material for: SIV reservoir depletion and clearance from neonatal macaques following transient CCR5 depletion
Source: Nat Microbiol. 2026 Oct 19;11(11):3102–14. doi: 10.1038/s41564-026-02511-3 (PMC13739729; doi:10.1038/s41564-026-02511-3)
Supplement: Supplementary file 1 [file 41564_2026_2511_MOESM1_ESM.pdf]

nature portfolio

Corresponding author(s): Dennis Hartigan-O'Connor

Last updated by author(s): 6/18/2026

# Reporting Summary

Nature Portfolio wishes to improve the reproducibility of the work that we publish. This form provides structure for consistency and transparency in reporting. For further information on Nature Portfolio policies, see our Editorial Policies and the Editorial Policy Checklist.

## Statistics

For all statistical analyses, confirm that the following items are present in the figure legend, table legend, main text, or Methods section.

| n/a | Confirmed | |
|---|---|---|
| ☐ | ☒ | The exact sample size ($n$) for each experimental group/condition, given as a discrete number and unit of measurement |
| ☒ | ☐ | A statement on whether measurements were taken from distinct samples or whether the same sample was measured repeatedly |
| ☐ | ☒ | The statistical test(s) used AND whether they are one- or two-sided<br>*Only common tests should be described solely by name; describe more complex techniques in the Methods section.* |
| ☐ | ☒ | A description of all covariates tested |
| ☐ | ☒ | A description of any assumptions or corrections, such as tests of normality and adjustment for multiple comparisons |
| ☐ | ☒ | A full description of the statistical parameters including central tendency (e.g. means) or other basic estimates (e.g. regression coefficient) AND variation (e.g. standard deviation) or associated estimates of uncertainty (e.g. confidence intervals) |
| ☐ | ☒ | For null hypothesis testing, the test statistic (e.g. $F$, $t$, $r$) with confidence intervals, effect sizes, degrees of freedom and $P$ value noted<br>*Give P values as exact values whenever suitable.* |
| ☒ | ☐ | For Bayesian analysis, information on the choice of priors and Markov chain Monte Carlo settings |
| ☒ | ☐ | For hierarchical and complex designs, identification of the appropriate level for tests and full reporting of outcomes |
| ☒ | ☐ | Estimates of effect sizes (e.g. Cohen's $d$, Pearson's $r$), indicating how they were calculated |

*Our web collection on statistics for biologists contains articles on many of the points above.*

## Software and code

Policy information about availability of computer code

| | |
|---|---|
| Data collection | No software was used. |
| Data analysis | R version 4.5.0 (2025-04-11) |

For manuscripts utilizing custom algorithms or software that are central to the research but not yet described in published literature, software must be made available to editors and reviewers. We strongly encourage code deposition in a community repository (e.g. GitHub). See the Nature Portfolio guidelines for submitting code & software for further information.

## Data

Policy information about availability of data

All manuscripts must include a data availability statement. This statement should provide the following information, where applicable:

- Accession codes, unique identifiers, or web links for publicly available datasets
- A description of any restrictions on data availability
- For clinical datasets or third party data, please ensure that the statement adheres to our policy

All data supporting the findings of this study are available within the paper and its Supplementary Information, or are available from the corresponding author upon request.

## Research involving human participants, their data, or biological material

Policy information about studies with human participants or human data. See also policy information about sex, gender (identity/presentation), and sexual orientation and race, ethnicity and racism.

| | |
|---|---|
| Reporting on sex and gender | No human participants or human data |
| Reporting on race, ethnicity, or other socially relevant groupings | No human participants or human data |
| Population characteristics | No human participants or human data |
| Recruitment | No human participants or human data |
| Ethics oversight | No human participants or human data |

Note that full information on the approval of the study protocol must also be provided in the manuscript.

# Field-specific reporting

Please select the one below that is the best fit for your research. If you are not sure, read the appropriate sections before making your selection.

☒ Life sciences ☐ Behavioural & social sciences ☐ Ecological, evolutionary & environmental sciences

For a reference copy of the document with all sections, see nature.com/documents/nr-reporting-summary-flat.pdf

# Life sciences study design

All studies must disclose on these points even when the disclosure is negative.

| | |
|---|---|
| Sample size | Sample size was determined using pwr.t.test to calculate the group size required for 80% power to detect a 1.8-fold standardized mean difference in time to rebound. |
| Data exclusions | No data were excluded from the analyses. |
| Replication | As of the time of publication, no measures were taken to reproduce the findings. |
| Randomization | Allocation into experimental groups was random. |
| Blinding | Investigators were not blinded to group allocation. |

# Reporting for specific materials, systems and methods

We require information from authors about some types of materials, experimental systems and methods used in many studies. Here, indicate whether each material, system or method listed is relevant to your study. If you are not sure if a list item applies to your research, read the appropriate section before selecting a response.

### Materials & experimental systems

| n/a | Involved in the study | |
|---|---|---|
| ☐ | ☒ | Antibodies |
| ☐ | ☒ | Eukaryotic cell lines |
| ☒ | ☐ | Palaeontology and archaeology |
| ☐ | ☒ | Animals and other organisms |
| ☒ | ☐ | Clinical data |
| ☒ | ☐ | Dual use research of concern |
| ☒ | ☐ | Plants |

### Methods

| n/a | Involved in the study | |
|---|---|---|
| ☒ | ☐ | ChIP-seq |
| ☐ | ☒ | Flow cytometry |
| ☒ | ☐ | MRI-based neuroimaging |

## Antibodies

| | |
|---|---|
| Antibodies used | Anti-CCR5/CD3 bispecific antibody clones 5C7/SP34 was provided by the NIH Nonhuman Primate Reagent Resource (no catalog number). Anti-CD4 clone CD4R1 was provided by the NIH Nonhuman Primate Reagent Resource (#AB_2716322). Anti-CD28 clone CD28.2 was provided by the NIH Nonhuman Primate Reagent Resource (#AB_2819274). Anti-CD49d clone L25 was provided by BD |

| | |
|---|---|
| | Biosciences (#340976). Anti-CD3 clone SP34-2 was provided by BD Biosciences (#557917). Anti-CD4 clone L200 was provided by BD Biosciences (#551980). Anti-CD8 clone SK1 was provided by BD Biosciences (#565310). Anti-CD95 clone DX2 was provided by BD Biosciences (#555671). Anti-CCR7 clone 3D12 was provided by BD Biosciences (#560922). Anti-HLA-DR clone G46-6 was provided by BD Biosciences (#556642). Anti-CCR5 clone 3A9 was provided by BD Biosciences (#550856). Anti-TNF-alpha clone MAB11 was provided by BD Biosciences (#557996). Anti-IFN-gamma clone B27 was provided by BD Biosciences (#557643). Anti-CD8 clone MT807R1 was provided by NIH Nonhuman Primate Reagent Resource (#AB_2716320). Anti-CD11c clone N418 was provided by BD Biosciences (#565592). Anti-CD11b clone ICRF44 was provided by BD Biosciences (#555386). Anti-CD14 clone M5E2 was provided by BD Biosciences (#557152). Anti-CD16 clone 3G8 was provided by BD Biosciences (#557920). Anti-CD20 clone 2H7 was provided by BD Biosciences (#556631). Anti-CD25 clone M-A251 was provided by BD Biosciences (#557138). Anti-CD80 clone L307.4 was provided by BD Biosciences (#557226). Anti-CD159 clone REA110 was provided by Miltenyi (#130-122-329). Anti-CD185 clone SPRCL5 was provided by ThermoFisher (#12-7185-82). Anti-CD279 clone EH12.2H7 was provided by ThermoFisher (#MA5-28584). |
| Validation | Validation of all antibodies for use with macaque cells was stated on the manufacturer's product page and confirmed in the reactivity database at https://www.nhpreagents.org/ReactivityDatabase. Validation of the rhesusized CCR5/CD3 bispecific antibody was provided by the manufacturer, the NIH Nonhuman Primate Reagent Resource; the approach to validation was published in DOI 10.1089/aid.2017.0159. |

## Eukaryotic cell lines

Policy information about cell lines and Sex and Gender in Research

| | |
|---|---|
| Cell line source(s) | NIH/NIAID Reagent Resource (BEI Resources) |
| Authentication | The 174xCEM cells were not further authenticated. |
| Mycoplasma contamination | The received cell lines were not tested for Mycoplasma contamination. |
| Commonly misidentified lines (See ICLAC register) | None used |

## Animals and other research organisms

Policy information about studies involving animals; ARRIVE guidelines recommended for reporting animal research, and Sex and Gender in Research

| | |
|---|---|
| Laboratory animals | Rhesus macaques (Macaca mulatta) |
| Wild animals | *Provide details on animals observed in or captured in the field; report species and age where possible. Describe how animals were caught and transported and what happened to captive animals after the study (if killed, explain why and describe method; if released, say where and when) OR state that the study did not involve wild animals.* |
| Reporting on sex | Sex was considered in the study design. There was no randomization by sex. The sexes of the animals used are given in Ext. Data Table 1. Sex-based analysis was performed but no significant effect of sex was detected. |
| Field-collected samples | *For laboratory work with field-collected samples, describe all relevant parameters such as housing, maintenance, temperature, photoperiod and end-of-experiment protocol OR state that the study did not involve samples collected from the field.* |
| Ethics oversight | This study was approved in advance by the University of California, Davis (UC Davis) Institutional Animal Care and Use Committee (IACUC). |

Note that full information on the approval of the study protocol must also be provided in the manuscript.

## Plants

| | |
|---|---|
| Seed stocks | *Report on the source of all seed stocks or other plant material used. If applicable, state the seed stock centre and catalogue number. If plant specimens were collected from the field, describe the collection location, date and sampling procedures.* |
| Novel plant genotypes | *Describe the methods by which all novel plant genotypes were produced. This includes those generated by transgenic approaches, gene editing, chemical/radiation-based mutagenesis and hybridization. For transgenic lines, describe the transformation method, the number of independent lines analyzed and the generation upon which experiments were performed. For gene-edited lines, describe the editor used, the endogenous sequence targeted for editing, the targeting guide RNA sequence (if applicable) and how the editor was applied.* |
| Authentication | *Describe any authentication procedures for each seed stock used or novel genotype generated. Describe any experiments used to assess the effect of a mutation and, where applicable, how potential secondary effects (e.g. second site T-DNA insertions, mosiacism, off-target gene editing) were examined.* |

## Flow Cytometry

### Plots

Confirm that:

☒ The axis labels state the marker and fluorochrome used (e.g. CD4-FITC).

☒ The axis scales are clearly visible. Include numbers along axes only for bottom left plot of group (a 'group' is an analysis of identical markers).

☒ All plots are contour plots with outliers or pseudocolor plots.

☒ A numerical value for number of cells or percentage (with statistics) is provided.

### Methodology

| | |
|---|---|
| Sample preparation | PBMC were separated from EDTA-anticoagulated whole blood using Ficoll-Hypaque. |
| Instrument | BD FACSymphony |
| Software | FlowJo |
| Cell population abundance | No sorting was performed. |
| Gating strategy | The gating strategy employed began with a preliminary FSC/SSC lymphocyte/mononuclear cell gate. For T-cell gating we then used a live CD3+ gate, followed by either CD4+CD8- or CD8+CD4- gates. Within those gates, memory cells were identified as CD95+; or effector-memory (EM) CD8+ T cells were identified as CD95+CCR7-. For APC gating we used a live CD3- gate and identified HLA-DR+ cells within it. Boundaries between "negative" and "positive" staining populations were set using FMO controls. |

☒ Tick this box to confirm that a figure exemplifying the gating strategy is provided in the Supplementary Information.
